# Complete Genome Sequence of Senecavirus A Strain SVV HN16 Identified in China

**DOI:** 10.1128/genomeA.01168-17

**Published:** 2017-11-02

**Authors:** Tianxia Luo, Shuaifei Xu, Jingfeng Xiong, Danping Su, Dongsheng He

**Affiliations:** College of Veterinary Medicine, South China Agricultural University, Guangzhou, Guangdong, People’s Republic of China

## Abstract

A virus that caused blisters and ulcers in pigs in China was detected to be a strain of Senecavirus A (SVA). Complete genome sequencing and analysis results showed that the isolate shares 93.8% to 99.1% identity with other isolates that have been reported, proving that a new isolate of SVA was found in China.

## GENOME ANNOUNCEMENT

Senecavirus A (SVA) belongs to the genus *Senecavirus* and family *Picornaviridae* and is closely related to members of the genus *Cardiovirus*. It is a nonenveloped, single-stranded, positive-sense RNA virus (1). Piglets infected with SVV always appear lame, and blisters and ulcers caused by the virus may lead to death (2). The virus was originally isolated in 1998 (3) but was not taken seriously until a large-scale outbreak in Brazil in 2014 (4, 5). The disease was first reported in China in 2016 (6).

In March 2016, a similar outbreak causing blisters and ulcers occurred on a swine farm in south China. Samples were collected, and a one-step reverse transcription-PCR kit (TaKaRa, Japan) was used to identify the pathogen. The result showed that it was a strain of SVA and not a strain of foot-and-mouth disease virus, swine vesicular disease virus, or vesicular stomatitis virus. This strain was named SVV HN16 (GenBank accession no. MF893200). To study the genomics of this SVA isolate, the complete genome was amplified by means of segmented cloning. Then, the target fragments were purified, inserted into pMD19-T vectors, and sequenced by Ruibio Biotech in Beijing, China.

The sequencing results suggested that isolate SVV HN16 consisted of 7,280 bp, in which the 5′ untranslated region (UTR) contained 668 bp and the 3′ UTR, excluding the polyA tail, contained 66 bp. Moreover, an open reading frame contained 6,546 bp, encoding a polyprotein with 2,181 amino acids. Homology analysis with the DNASTAR Lasergene 7 package found that the homology between this strain and other isolates from Thailand (GenBank accession no. KY368743 and KY368744), Canada (KY486161 and KY486166), China (KX173338 and KT321458), Brazil (KR063107 and KR063108), and the United States (DQ641257 and KT757280) was between 93.8% and 99.1%; the homology of the polyproteins was between 97.8% and 99.8%, confirming that a new isolate of SVA was found in China.

### Accession number(s).

The whole-genome sequence of SVA strain SVV HN16 has been deposited in GenBank under the accession number MF893200.
